# The role of virtual reality in balance training for Parkinson's disease: a systematic review and meta-analysis

**DOI:** 10.3389/fresc.2026.1768929

**Published:** 2026-05-25

**Authors:** Sudhindra Vooturi, Sai Sirisha, Sumanth Panem, Monica Yazala

**Affiliations:** 1 Department of Rehabilitation, Krishna Institute of Medical Sciences, Secunderabad, Telangana, India; 2 Department of General Medicine, Dr. D. Y. Patil Medical College, Hospital and Research Centre, Pune, Maharashtra, India

**Keywords:** balance training, Berg Balance Scale (BBS), Parkinson's, task training, virtual rehab

## Abstract

**Background and objectives:**

The use of virtual reality (VR) as a therapeutic modality to improve balance in patients with Parkinson's disease (PD) is promising due to its interactive simulations. However, the advantage of VR over conventional balance training has yet to be established. This systematic review and meta-analysis evaluates and summarizes randomized controlled trials (RCTs) comparing VR interventions to conventional physiotherapy.

**Materials and methods:**

A comprehensive search of the PubMed, EBSCOHost, and SciELO databases was performed. Article selection and filtering were performed using Rayyan software.

**Results:**

The search yielded 185 research articles, of which 162 remained after removing duplicates. Screening the titles and abstracts for the use of VR and reviewing the full-texts for the RCT design yielded 17 articles, six of which used the Berg Balance Scale (BBS). The data from these remaining six trials were included in this systematic review. Data extraction resulted in 214 participants (105 controls and 109 in the VR intervention). The average age of the participants was 61.43 ± 13.3 years, with 74 (34.6%) women and a mean PD duration of 7.11 ± 1.91 years. The average number of sessions was 25 (10–60), with an average follow-up period of 3.5 months. At follow-up, the control group improved by an average of 1.64 ± 1.84 points on the BBS, whereas the VR group improved by an average of 2.85 ± 1.74 points. There was no significant difference (*p* = 0.269) between the groups for the absolute change on the BBS.

**Conclusions:**

VR-based interventions for patients with PD are as effective as conventional physiotherapy for improving balance in short-term follow-ups. VR-based interventions alongside conventional physiotherapy may deliver efficient and tailor-made balance training.

## 1 Introduction

Parkinson's disease (PD) progressively impairs motor functions, such as balance, leading to a significant increase in the risk of falls and resulting in a diminished quality of life (1). Nearly two-thirds of individuals with PD report at least one fall every year, and 39% of these falls are recurrent (2). The risk of falls in patients with PD is alarming, considering they have an average 3.5 times higher chance of hip fracture than age-matched healthy adults (3). Although a detailed motor examination is preferable to self-reported fall diaries, an ideal clinical balance scale should be multi-dimensional yet provide detailed information regarding one domain, such as the risk of falling and potential mechanisms of falls (4). The Berg Balance Scale (BBS) is a validated and reliable scale to assess postural control in patients with PD. Often considered the “gold standard” balance scale, the BBS has limitations in assessing reactive postural control and has a ceiling effect (5). Importantly, the BBS has the ability to predict recurrent from non-recurrent fallers with an accuracy of 0.79 in area under curve (AUC). Furthermore, the BBS can also differentiate future fallers from non-fallers (6).

Schlenstedt C et al. (7) reported the findings of a comparative study on the use of three balance scales—the Fullerton Advanced Balance scale (FAB), the Mini-Balance Evaluation Systems Test (BESTest), and the BBS—in subjects with PD. The authors reported that, in patients with PD, the FAB scale correctly predicted 70% of fallers, whereas the BBS and Mini-BESTest scales identified 65% and 50% of fallers, respectively. However, the BBS was better at predicting true non-fallers (70%) than the FAB scale (60%). The study concluded that the likelihood of being a faller increases 1.6-, 1.7-, and 1.9-fold in case of a positive test result on the FAB scale, Mini-BESTest, and BBS, respectively (7).

Advancements in virtual reality (VR) as a physical therapeutic modality to improve balance in patients with PD are surpassing the conventional therapies (8). The existing literature on the role of VR in balance training for patients with PD shows promising results due to its interactive simulations of real-life scenarios in a safe and controlled space (9). Despite being a promising intervention, the advantages of VR over conventional balance training have yet to be established. The current literature on the effectiveness of VR over conventional balance training is limited by a lack of randomization, small sample sizes, and variable study designs and interventions.

This systematic review and meta-analysis aims to evaluate and summarize the findings of randomized controlled trials (RCTs) comparing VR interventions to conventional physiotherapy using practical outcome scales, such as the BBS, in subjects with PD.

## 2 Methods

### 2.1 Design

A systematic review was conducted in accordance with the Preferred Reporting Items for Systematic Reviews and Meta-Analyses (PRISMA) guidelines (10). Relevant randomized controlled trials were searched for in the following databases: PubMed, EBSCOHost, and Web of Science.

### 2.2 Research strategy

Searches were performed using a combination of terms following the PICOS strategy (11) from the English MeSH thesaurus using a combination of terms that included “Virtual Reality”, “Virtual Reality Therapy”, “Task-specific Virtual Reality”, and “Parkinson's Disease” and/or “Parkinsonism”. The searched keywords had to appear in the titles, abstracts, and/or keywords of the results from the databases.

### 2.3 Eligibility criteria

Randomized controlled clinical trials were included if they used VR or Augmented Reality (AR)-based training for balance correction as an intervention compared to other nonpharmacological exercise interventions or controls. The articles had to be written in English and published in peer-reviewed, indexed journals by September 2025. Identification of the likelihood of falling is an important step in the prevention of falls, and the purpose of gait and balance training is to prevent falls. Additionally, the BBS, often considered the gold standard for balance assessment, better predicts the likelihood of falls (7). Therefore, we only included studies that used the Berg Balance Scale to assess the primary outcome.

Exclusion criteria included studies in which VR and/or AR were either combined or compared with therapies other than exercise. Additionally, studies that combined different types of neurological diseases other than PDs were also excluded. Moreover, studies that did not use the Berg Balance Scale were also excluded.

### 2.4 Study selection process

Relevant articles were identified through a bibliographic search of the aforementioned databases. Duplicate removal and study eligibility screening were carried out in two phases using Rayyan software (12). The article selection process consisted of two phases. Phase 1: Initial Screening was performed by two independent authors (each from a different institution) by reading the titles, abstracts, and/or keywords. During Phase 2, the full-length texts of the articles included after Phase 1 were assessed to determine their eligibility. During Phase 2, atleast 3 out of 4 authors (SV, SS, MY, SP) should agree for the inclusion of the study for analysis.

### 2.5 Data extraction

Using the PICOS strategy (11), two of the authors extracted essential data into an Excel spreadsheet, followed by subsequent verification by the third author. The data extracted included age, gender distribution, severity of PD, duration of PD, sample size (for the entire study and for individual groups), intervention details (duration, frequency, intensity, and type), follow-up duration, follow-up losses, dropouts, and the difference in BBS scores between the comparative study arms.

- Intervention: Virtual Reality-Based Exercise.
- Control: Conventional Physiotherapy.
- Outcome: Berg Balance Scale.

### 2.6 Risk-of-Bias assessment tool

Publication bias was assessed using the Cochrane Risk of Bias (RoB 2) tool, wherein the judgment for each bias domain (randomization process, timing of identification or recruitment, deviation from intended interventions, missing outcome data, measurement of outcome, and selection of reported results) was low, as was the overall risk of bias.

### 2.7 Treatment effect analysis

The difference in mean change with intervention between the VR and conventional groups was extrapolated from the studies by two independent authors. A *p* < 0.05 was considered statistically significant. Heterogeneity among the studies is reported visually as forest plots, with statistics reported as chi-square tests and I² statistics. All statistical analyses were performed using metaanalysisonline, A5 Genetics Ltd.

## 3 Results

### 3.1 Study selection and general characteristics

**Figure 1:**
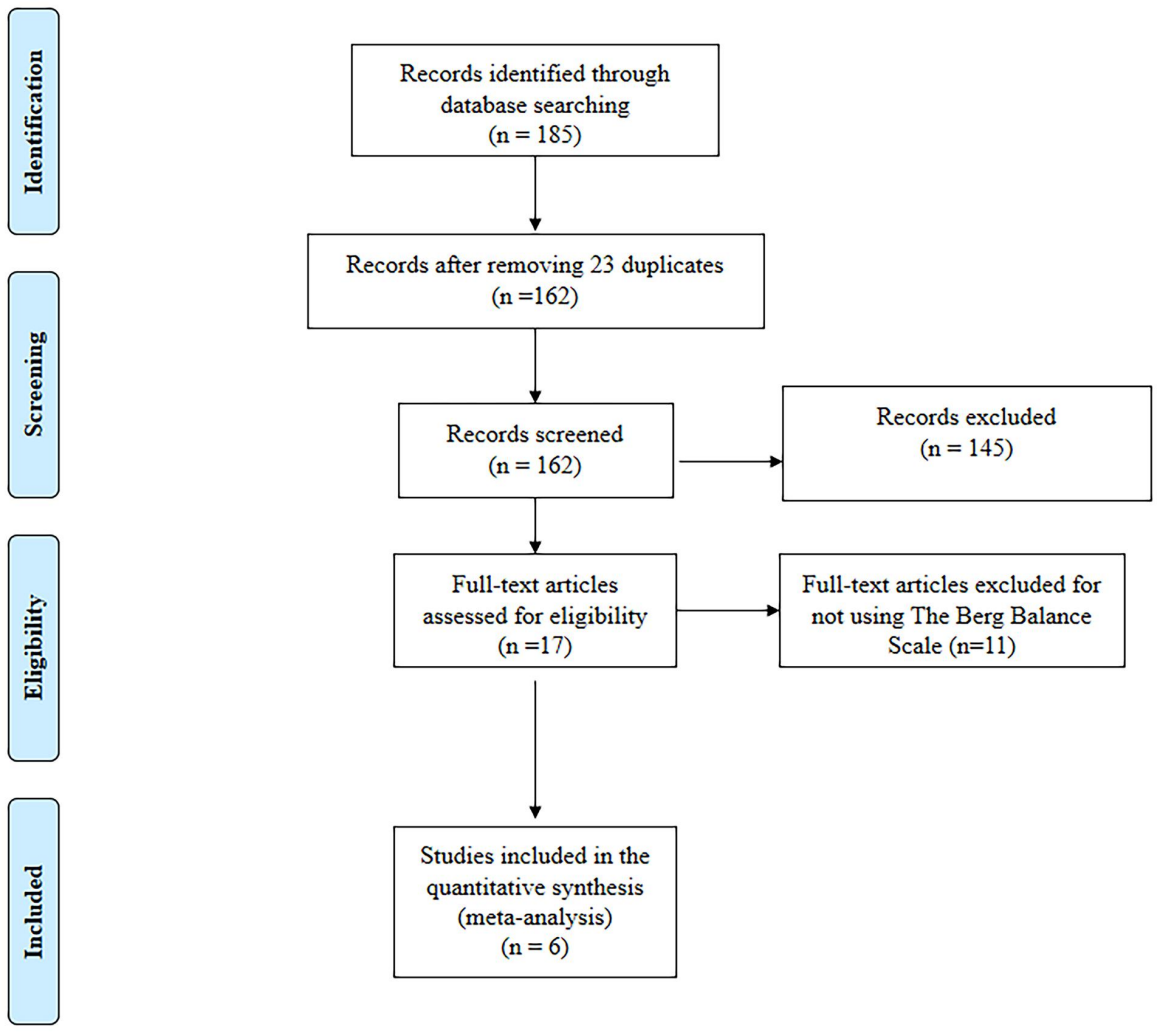
PRISMA flowchart of the selection of studies.

Of the 185 research articles retrieved from the databases, 162 remained after removing duplicates. After screening the titles and abstracts for the use of VR and reviewing the full-texts for the RCT design, 17 articles remained, six of which were RCTs that used the BBS. The data from these six trials were included in this systematic review. Figure 1 summarizes the flowchart of the study selection. Of these six RCTs, one was conducted in the Netherlands, [van den Heuvel MR et al. (13)], one in the Republic of Korea (Lee NY et al. (14)), one in Taiwan [Yang WC et al. (15)], one in Italy [Gandolfi M et al. (16)], one in China [Feng H et al. (17)] and one in Türkiye [Gulcan K et al. (18)], representing a mix of literature from both the East and West.

### 3.2 Sample characteristics

Table 1 summarizes the findings of this systematic review, which included a total of 214 subjects (105 controls and 109 in the VR intervention) diagnosed with Parkinson's disease and examined while at Stages II or III on the modified Hoehn and Yahr stages. The average age of the participants was 61.43 ± 13.3 years, with 74 of the patients (34.6%) being women, and a mean duration of PD of 7.11 ± 1.91 years.

**Table 1:** Summary of the studies included in the data extraction.

S. No.	Publication Year	First Author	Age (years)	Women (number)	Severity of PD	Duration of PD (years)	Control	Intervention Sample	Control Therapy	Intervention	No. of Sessions	Events	Delta Control BBS	Delta Intervention BBS	Dropouts	Follow-up (months)
1	2013	van den Heuvel MR (13)	67.5	13	2 & 3		14	17	Conventional	AR + VR	2 sessions per week×5 weeks	0	−1	1	2 in the control group	Pre; Post; 3 Months; 12 Months
2	2015	Lee NY (14)	69.2	10			10	10	NDT + FES	Nintendo Wii + FES	5 sessions per week×6 weeks		0.04	2.1		1.5
3	2016	Yang WC (15)	35	9	2 & 3	8.8	12	11	Conventional	VR	2 sessions per week×6 weeks	0	2.83	2.73	1 in the control grou*p* + 2 in the intervention group due to loss of interest	2
4	2017	Gandolfi M (16)	68.6	25	2 & 3	6.8	38	38	Sensory Integration	Home-Based VR	3 sessions per week×7 weeks	0	4.05	3.21	0	1
5	2019	Feng H (17)	68.3	13	3	6.8	14	14	Conventional	VR-Based Games	5 sessions per week×12 weeks	0	1.93	6.07	0	3
6	2023	Gulcan K (18)	60	4	3	6	17	17	Conventional	AR & VR	3 sessions per week×6 weeks	0	2	2	2 per group (voluntary withdrawal)	1.5

PD,Parkinson's Disease; VR,Virtual Reality; AR,Augmented Reality; BBS,Berg Balance Scale.

In all the studies, the intervention arm was game-based VR therapy, and the control group received conventional physical therapy. While Gandolfi M et al. (16) used a home-based VR model, Gulcan K et al. (18) utilized augmented reality in addition to VR. The average number of sessions was 25 (from 10 to 60). The dropout rate was similar between the groups: five (4.8%) in the control group vs. three (2.7%) in the VR intervention group. All dropouts reported were voluntary. The average follow-up period was 3.5 months, while van den Heuvel MR et al. (13) reported a follow-up of 12 months, and Gandolfi M et al. (16) reported the shortest follow-up period of one month.

**Figure 2:**
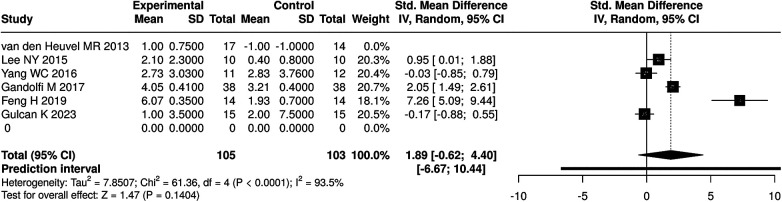
Forest plot illustrating standardized mean differences and confidence intervals from six studies comparing experimental and control groups, with overall effect estimate.

At follow-up, five studies reported improvement on the BBS scale in both the control and VR groups, except for van den Heuvel MR et al. (13), who reported deterioration on the BBS scale in their control group. On average, the control group improved by 1.64 ± 1.84 points on the BBS, whereas the VR group improved by 2.85 ± 1.74 points at follow-up. There was no significant difference (*p* = 0.269) between the study groups for the absolute change (delta between pre- and post-testing) on the BBS. Based on the analysis performed using a random effects model with an Inverse variance method to compare the standardized mean difference (SMD), there was no statistical difference between the two cohorts; the summarized standardized mean difference (SMD) was found to be 1.89, with a 95% confidence interval of −0.62 to 4.4. The test for the overall effect did not show a significant effect. However, a significant heterogeneity was detected (*p* < 0.01), suggesting inconsistent effects in magnitude and/or direction. The I² value indicated that 93% of the variability among studies arose from heterogeneity rather than random chance. Figure 2 summarizes the Forest plot comparing the BBS outcomes.

## 4 Discussion

In this review on the role of virtual reality (VR) in balance training for subjects with Parkinson's disease (PD), we report that although VR shows promise, it does not yet have a clear-cut advantage over conventional physiotherapy.

In the review, 65.5% of participants were men, confirming the propensity of men to constitute a larger proportion of individuals with PD (19). Similarly, we found an average age of 61.43 years, which is the typical age of PD onset (19).

The virtual reality (VR) treatments explored in the studies we included were predominantly non-immersive approaches to enhance balance in people with PD. While Lee NY et al. (14) employed the Nintendo Wii Balance Board for VR training, Yang et al. (15) and Van den Heuvel et al. (13) used wireless balance training boards, and Gandolfi et al. (16) adopted a home rehabilitation approach. Irrespective of the device used for training, aggregate improvement in the BBS was not greater than with conventional physiotherapy. Perhaps the pitfalls of BBS, such as the ambiguity of the scoring system, which requires the tester's judgment, and the lack of specificity regarding the use of compensatory strategies, may have influenced the scores. The blinding strategy used by the authors of the six RCTs in the current study is inconclusive (5). The MDC was determined using the difference in mean between the control and intervention groups for the BBS, incorporating the standard deviation. Importantly, MDC values vary depending on the population studied and the methodologies used by the different studies selected in this review.

In subjects with PD, the interaction between the somatosensory and proprioceptive systems is often compromised. Therefore, VR training with feedback is expected to increase the neuroplastic activation of these systems and lead to better results than conventional physiotherapy (20). However, we did not observe a clear advantage of VR therapy over conventional physiotherapy, probably due to variations in VR interventions. Perhaps conventional physiotherapy, with its direct, in-person supervision and nuanced feedback, still outperforms the haptic feedback loops of VR models (21). Moreover, conventional physiotherapy takes place in real-world contexts that are difficult to simulate. It is important to remember that, from a cognitive point of view, patients with PD and the geriatric population in general prefer the personal care and empathy of a human therapist rather than an isolating technology (21). Furthermore, the improvement in BBS scores in both groups was found to be less than the minimal detectable change of 4–7 points. Perhaps the duration of training was not sufficient; therefore, reviews focusing on long-term training are desirable.

### 4.1 Strengths and limitations

While the strengths of our review include improved sample power and precision, objectivity (trials that used the BBS were included), and the fact that analysis of RCTs is often considered a high level of evidence. We have taken precautions to minimize the inherent limitations of reviews, such as heterogeneity among the included studies, ecological fallacy, and oversimplification of conclusions.

## 5 Conclusion

This systematic synthesis of evidence from RCTs on the potential of VR-based interventions to improve balance in patients with PD is neither superior nor inferior to conventional physiotherapy. Importantly, VR-based interventions, in addition to conventional physiotherapy, may deliver efficient balance training that is tailored to individual needs. Future research could focus on the role of VR-based interventions in task-oriented balance training for subjects with PD.

## Data Availability

The data can be accessed upon submitting a reasonable request to the corresponding author.
